# Sodium iodate induces ferroptosis in human retinal pigment epithelium ARPE-19 cells

**DOI:** 10.1038/s41419-021-03520-2

**Published:** 2021-03-03

**Authors:** Binghua Liu, Weiyan Wang, Arman Shah, Meng Yu, Yang Liu, Libo He, Jinye Dang, Li Yang, Mengli Yan, Yuling Ying, Zihuai Tang, Ke Liu

**Affiliations:** 1 Key Laboratory of Bio-Resource and Eco-Environment of Ministry of Education, College of Life Sciences, Sichuan University, Chengdu, 610065 Sichuan PR China; 2 Laboratory of Molecular Biology, College of Medicine, Chengdu University, Chengdu, 610106 Sichuan PR China

**Keywords:** Disease model, Cell death

## Abstract

Sodium iodate (SI) is a widely used oxidant for generating retinal degeneration models by inducing the death of retinal pigment epithelium (RPE) cells. However, the mechanism of RPE cell death induced by SI remains unclear. In this study, we investigated the necrotic features of cultured human retinal pigment epithelium (ARPE-19) cells treated with SI and found that apoptosis or necroptosis was not the major death pathway. Instead, the death process was accompanied by significant elevation of intracellular labile iron level, ROS, and lipid peroxides which recapitulated the key features of ferroptosis. Ferroptosis inhibitors deferoxamine mesylate (DFO) and ferrostatin-1(Fer-1) partially prevented SI-induced cell death. Further studies revealed that SI treatment did not alter GPX4 (glutathione peroxidase 4) expression, but led to the depletion of reduced thiol groups, mainly intracellular GSH (reduced glutathione) and cysteine. The study on iron trafficking demonstrated that iron influx was not altered by SI treatment but iron efflux increased, indicating that the increase in labile iron was likely due to the release of sequestered iron. This hypothesis was verified by showing that SI directly promoted the release of labile iron from a cell-free lysate. We propose that SI depletes GSH, increases ROS, releases labile iron, and boosts lipid damage, which in turn results in ferroptosis in ARPE-19 cells.

## Introduction

The RPE layer consists of a single layer of neatly arranged and highly specialized cells located between the retinal photoreceptors and the choroid. The microvilli at the top of the RPE protrudes to the photoreceptor layer and envelops the outer segment of photoreceptors^1^. RPE cells have multiple physiological roles in forming the blood–retinal barrier to protect the neuroretina, nourish photoreceptor cells, remove dead cells, protect the retina from oxidative stress, secret growth factors, and more^2–5^. Therefore, the degeneration, injury, or death of RPE cells inevitably impairs visual function and may cause permanent blindness since mammalian RPE cells do not regenerate^6^.

Oxidative stress can increase the level of ROS^7^, deplete ATP^8^, and promote plasma membrane leakage^8^, DNA damage^9^, and premature aging^10^ in RPE cells. These lesions in turn obstruct the functionality of RPE cells, which leads to retinal degeneration. Therefore, many oxidative agents have been used to study the mechanism of retinal degeneration and evaluate treatments for retina protection. Among these, NaIO_3_, sodium iodate (SI) has been shown to be toxic and cause the death of retina cells in many mammals^11–14^. Despite the wide usage of SI to generate retinal degeneration models, the mechanism of SI-induced death of RPE cells is poorly understood. It was reported that SI treatment of RPE cells resulted in the aggregation of receptor-interacting protein kinase 3 (RIPK3) and the release of high-mobility group box 1 proteins (HMGB1)^15^. In addition, SI-induced cell death could be rescued by RIPK1 inhibitor necrostatin-1 (Nec-1) and RIPK3 inhibitor GSK′872, suggesting that necroptosis may be involved^15^. However, the detailed mechanism of SI-induced necroptosis is not clear. It is also unknown if other regulated cell death (RCD) signaling pathways are involved.

RCD is characterized by the requirement of dedicated molecular machinery for targeted elimination of damaged or injured cells^16^. Recently it has been demonstrated that ferroptosis, a form of RCD mediated by iron and lipid peroxidation, plays an important role in tert-butyl hydroperoxide (tBH)-induced RPE cell death^17^. Ferroptosis is controlled by glutathione peroxidase 4 (GPX4)^18,19^, or ferroptosis suppressor protein 1 (FSP1)^20,21^ which works to reduce lipid peroxidation. The mechanisms of ferroptosis have been revealed in many pathological processes by employing ferroptosis-inducing agents including erastin^22,23^, sulfasalazine^24^, sorafenib^25^, RSL3 (ref. ^26^), FIN56 (ref. ^27^), and more^28^. Accumulating evidence demonstrates that ferroptosis is a potential cause of many human disorders such as Alzheimer’s disease^29^, Huntington’s disease^30^, Parkinson’s disease^31^, and age-related macular degeneration (AMD)^17^. Although recent studies have revealed that oxidative stress-induced ferroptosis in RPE^7,17^, questions remain regarding the relationship between the RPE cell death induced by SI and ferroptosis.

To investigate the potential involvement of ferroptosis in SI-induced death of RPE cells, we herein examined the necrotic features of cultured ARPE-19 cells treated by SI. We found that SI induced a necrotic morphotype in ARPE-19 cells with the increase of labile iron level, ROS generation, oxidation of unsaturated fatty acids, and exhaustion of GSH and cysteine, suggesting that ferroptosis plays an important role in SI-induced damage and death of RPE cells.

## Results

### Apoptosis and necroptosis are not the only causes of cell death induced by SI

**Fig. 1:**
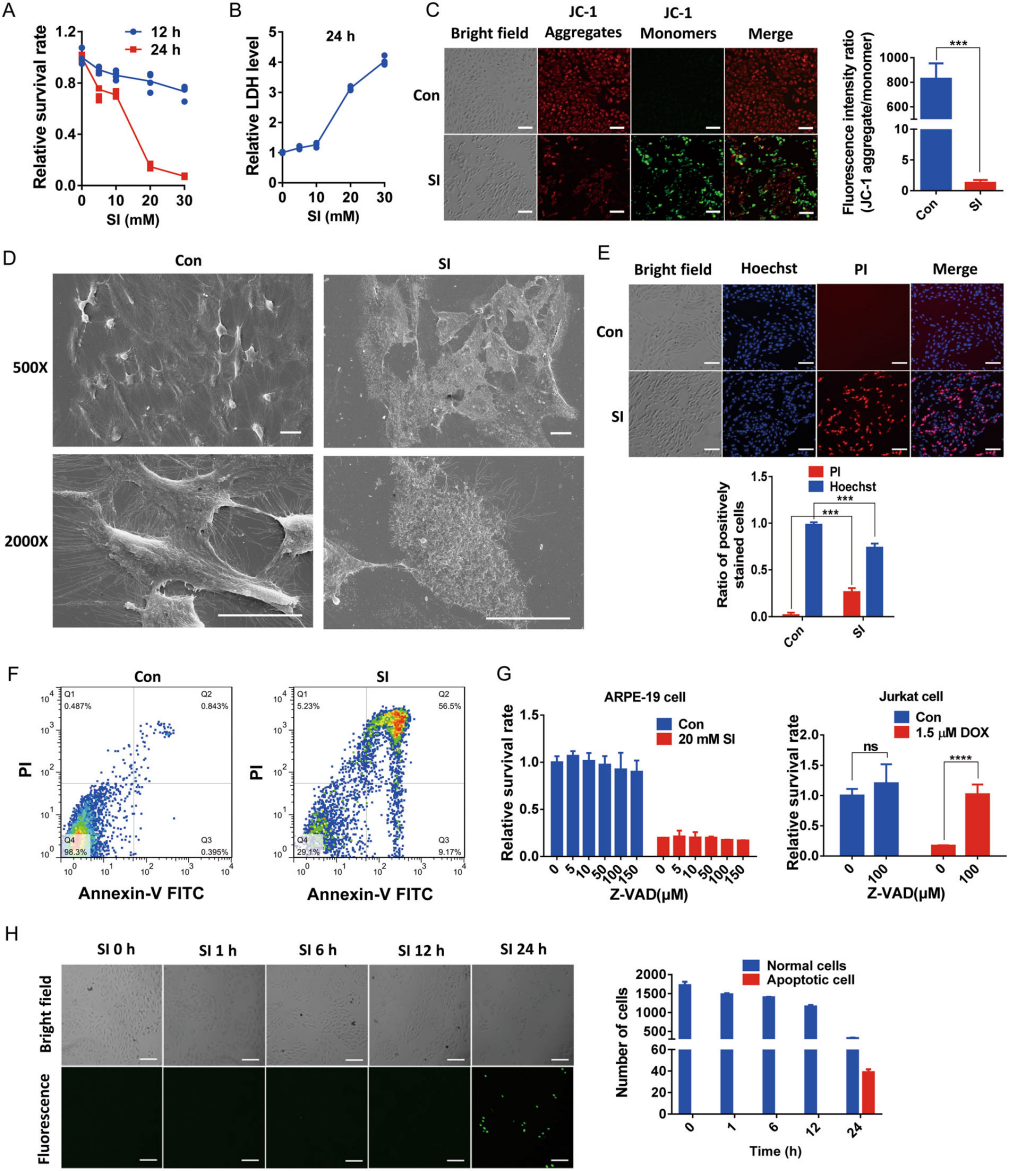
ARPE-19 cells treated with SI manifest necrotic phenotype. **A** Cell viabilities determined by CCK assays after NaIO_3_ treatment for 12 h or 24 h at indicated concentrations (*n* = 4). **B** ARPE-19 cells were cultured with NaIO_3_ at indicated concentrations for 24 h. After the treatment, the LDH levels were determined by the LDH assays (*n* = 4). **C** Left: Fluorescence images of cells stained with JC-1, an indicator of mitochondrial membrane potential (MMP). ARPE-19 cells were treated with 10 mM NaIO_3_ for 24 h. Scale bar: 100 μm. Right: The ratio of red/green fluorescence intensity shows the change of MMP (*n* = 3). **D** ARPE-19 cells treated with 10 mM NaIO_3_ for 24 h were observed by scanning electron microscope. Scale bar: 20 μm. **E** Upper: Fluorescence images of cells treated with 10 mM NaIO_3_ for 24 h followed by Hoechst 33342 and PI double staining. Scale bar: 100 μm. Lower: Cells positively stained with Hoechst and PI were counted using Image J software. Graphs represent the percentage of Hoechst and PI stained cells against total cells (*n* = 3). **F** Fluorescence-activated cell sorting (FACS) histograms of Annexin V-FITC/PI stained cells with (right) and without (left) the treatment of 10 mM NaIO_3_ for 24 h (Q1: cellular debris or necrotic cells; Q2: necrotic or late apoptotic cells; Q3: early apoptotic cells; Q4: viable cells). **G** Left: Effect of the caspase inhibitor Z-VAD on ARPE-19 cell death. Cells were pretreated with Z-VAD or solvent alone (DMSO) at indicated concentrations for 3 h and followed by co-treatment with or without 20 mM NaIO_3_ for 24 h (*n* = 4). Right: A positive control for apoptosis inhibition by Z-VAD. Jurkat cells were pretreated with 100 μM Z-VAD or solvent alone (DMSO) for 3 h followed by co-treatment with 1.5 μM doxorubicin (DOX) for 18 h (*n* = 4). **H** Left: TUNEL staining of apoptotic ARPE-19 cells. Cells were exposed to 10 mM NaIO_3_ for the indicated time. Scale bar: 100 μm. Right: numbers of apoptotic cells and normal cells in the microscope field of view were counted by image J software (*n* = 3).

At concentrations in the millimolar range that have been widely used for establishing degeneration models of retina^32^, we found that SI induced the death of ARPE-19 cells in a time and concentration-dependent manner (Fig. 1A, Fig. S1). The release of lactate dehydrogenase (LDH), which is a typical marker of cell death and plasma membrane disruption, increased with increasing concentrations of SI (Fig. 1B). In healthy control cells, the lipophilic dye JC-1 showed red fluorescence, indicating that mitochondria had intact mitochondrial membrane potential (MMP) (Fig. 1C, upper panels). In SI-treated cells, JC-1 was mostly in its monomeric green fluorescent form, suggesting the collapse of the MMP (Fig. 1C, lower panels). The damage of ARPE-19 cells induced by SI was also accompanied by the change of cell morphology. As shown by scanning electron microscopy, SI treatments resulted in significant losses of matrix structure and cell integrity (Fig. 1D). The loss of cell integrity caused by SI treatments was verified by robust propidium iodide (PI) staining, indicating disruption of the nuclear membrane (Fig. 1E). Although the loss of MMP can be induced by apoptosis, the similarity of Hoechst 33342 staining between control and SI-treated cells suggests that SI did not enhance apoptosis of ARPE-19 cells (Fig. 1E). The flow cytometric analysis of apoptotic cells revealed that after SI exposure only a small portion of cells was FITC Annexin V positive and PI negative (early apoptotic) and most of the cells were FITC Annexin V and PI-positive (end-stage apoptotic or necrotic) (Fig. 1F). Furthermore, the pan-caspase inhibitor Z-VAD did not alleviate cell death caused by SI, although it effectively inhibited doxorubicin-induced apoptosis of Jurkat cell (Fig. 1G). TUNEL staining also indicated that only a small portion of ARPE-19 cells was apoptotic after 24 h of SI treatment which caused a significant decrease in cell number (Fig. 1H). These data indicate that the SI-induced death of ARPE-19 cells is not apoptotic death. Instead, the involvement of membrane rupture and release of cytoplasmic contents suggests the death process is more necrotic.

**Fig. 2:**
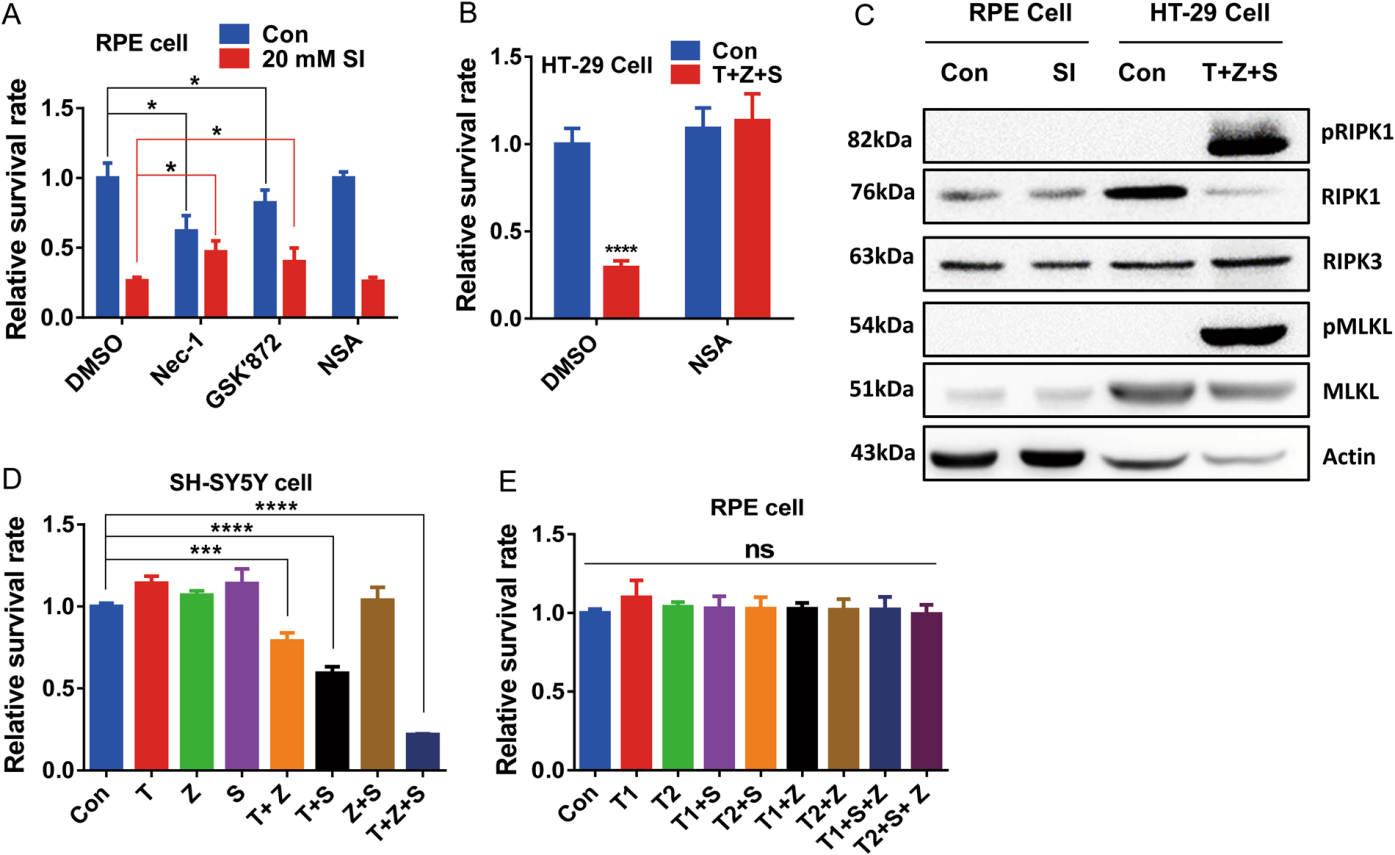
Necroptosis is not the main pathway related to the death of ARPE-19 cells induced by SI. **A** Cell viabilities of ARPE-19 cells treated with 200 μM Nec-1, 2 μM NSA or 10 μM GSK′872 for 24 h before 20 mM NaIO_3_ treatment for 24 h (*n* = 4). **B** HT-29 cells with or without necrosulfonamide (NSA) pretreatment was treated with the indicated necrosis-inducing agents for 16 h. T + Z + S: TNF-α (200 ng/ml), Z-VAD (20 μM), and SM-164 (10 μM) (*n* = 4). **C** Phospho-RIPK1 (S166), total RIPK1, total RIPK3, Phospho-MLKL (S358), and total MLKL were detected in ARPE-19 or HT-29 cells by western blotting. SI: 10 mM NaIO_3_ treatment for 24 h. T + Z + S: TNF-α (100 ng/ml), Z-VAD (20 μM), and SM-164 (10 μM) treatment for 3 h. **D** Cell viabilities of SH-SY5Y cells treated with the indicated necrosis-inducer agent(s) for 3 h. T: 100 ng/ml TNF-α; S: 10 μM SM-164; Z: 20 μM Z-VAD (*n* = 4). **E** Cell viabilities of ARPE-19 cells treated with the indicated necrosis-inducer agent(s) for 48 h. T1: 50 ng/ml TNF-α; T2: 100 ng/ml TNF-α; S: 10 μM SM-164; Z: 20 μM Z-VAD (*n* = 4).

It has been suggested that SI could induce necroptosis of ARPE-19 cells^15^. Necroptosis is a type of RCD that depends on a sequential activation of RIPK1, RIPK3, and MLKL^16,33^. To evaluate the involvement of necroptosis in SI-induced ARPE-19 cell death, cell viability was monitored in the presence of Nec-1, GSK′872 or NSA, inhibitors of RIPK1, RIPK3, and MLKL respectively, with or without SI (Fig. 2A). While Nec-1 and GSK′872 decreased cell viability in control cells without SI treatment, both inhibitors partially recovered the viability of SI-treated cells. However, the MLKL inhibitor NSA had no beneficial effects on SI-treated cells, although in a necroptosis model it robustly prevented the death of HT29 cells (compare Fig. 2A and Fig. 2B). The signaling of necroptosis involves many hallmarks including phosphorylation of RIPK1 (ref. ^34^) and MLKL^35^ which is required for the formation of necrosome. Although there was robust phosphorylation of RIPK1 and MLKL in the HT-29 cells necroptosis model, phosphorylation of RIPK1 and MLKL in SI-treated ARPE-19 cells was not detectable (Fig. 2C). ARPE-19 cells were also resistant to necroptosis induced by treatment of TNF-α/Z-VAD/SM-164, the best-characterized trigger for necroptosis as demonstrated in SH-SY5Y cells (Fig. 2D, E). These results suggest that necroptosis is unlikely to be the major pathway of SI-induced ARPE-19 cell death.

### SI-induced cell death is associated with iron homeostasis

**Fig. 3:**
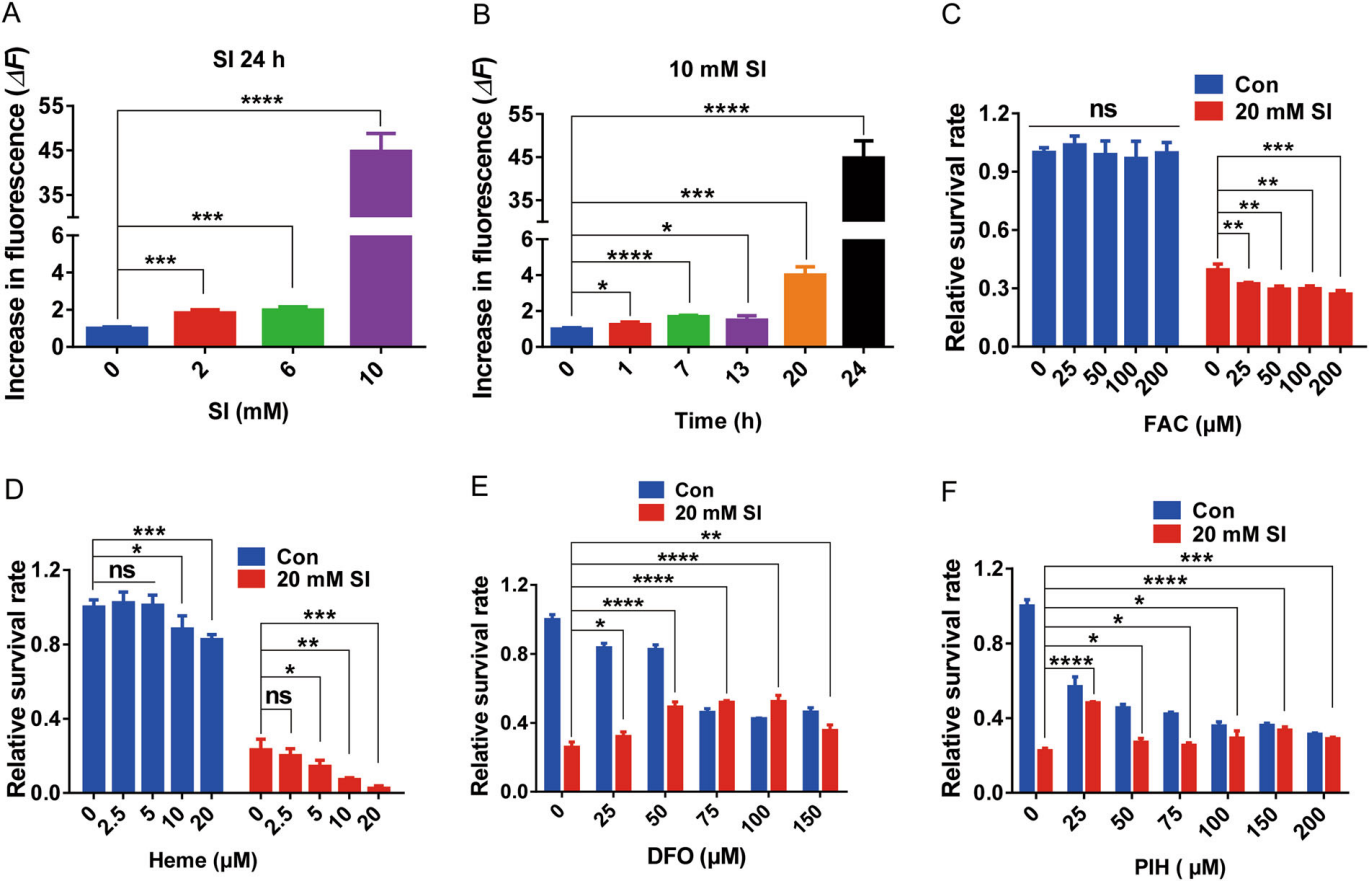
Death of ARPE-19 cells induced by SI is related to the intracellular labile iron. **A**, **B** Alteration of labile iron levels in ARPE-19 cells with NaIO_3_ treatment in concentration (**A**) or time (**B**) dependent manner. The labile iron levels were assessed as the increase in fluorescence (Δ*F*) 30 min after PIH addition (*n* = 4). **C** Viabilities of ARPE-19 cells treated with indicated concentrations of FAC (ammonium ferric citrate) for 24 h followed by additional 24 h treatment with or without 20 mM NaIO_3_ (*n* = 5). **D** Viabilities of ARPE-19 cells treated with indicated concentrations of Heme or vehicle alone (DMSO) for 24 h followed by additional 24 h treatment with or without 20 mM NaIO_3_ (*n* = 5). **E** The protective role of DFO for SI-induced cell death. ARPE-19 cells were pretreated with DFO at indicated concentrations or solvent alone (PBS) for 24 h prior to co-treatment with 20 mM NaIO_3_ for an additional 24 h (*n* = 4). **F** The protective role of PIH for SI-induced cell death. ARPE-19 cells were pretreated with PIH at indicated concentrations or solvent alone (DMSO) for 48 h prior to co-treatment with 20 mM NaIO_3_ for an additional 24 h (*n* = 4).

The necrotic morphotype of SI-induced ARPE-19 cell death without necrosome formation suggests the involvement of another form of necrosis. Ferroptosis, which is iron and ROS-dependent, has emerged as an important form of regulated necrosis implicated in various human diseases^36^. To investigate the possible involvement of ferroptosis in SI-induced ARPE-19 cell death, the relative labile iron pool (LIP) level in ARPE-19 cells was monitored by the calcein fluorescence quenching method under SI treatment. While SI treatment at lower concentrations or for shorter times moderately increased intracellular LIP, 10 mM NaIO_3_ treatment for 24 h boosted intracellular LIP about 45-fold (Fig. 3A, B). These data suggest that SI-induced cell death may correlate to increased intracellular LIP, although the intracellular LIP after NaIO_3_ treatment at more lethal levels cannot be monitored due to extensive cell death. Providing exogenous iron by adding FAC (ammonium ferric citrate) (Fig. 3C) or heme (Fig. 3D) into cell cultures enhanced SI-induced cell death. Conversely, chelating iron by treating with DFO, a widely used ferroptosis inhibitor^7^, decreased SI-induced death of APRE-19 cells or primary mouse RPE cells (Fig. 3E, Fig. S2). The decreased cell viability under DFO treatment without SI treatment as shown in Fig. 3E is likely due to the cytotoxicity of DFO^37^. In addition, using another iron chelator pyridoxal isonicotinoyl hydrazone (PIH), which is more membrane permeable than DFO, also partially protected cells from SI-induced death (Fig. 3F, Fig. S2).

### SI induces ROS production and lipid damage

**Fig. 4:**
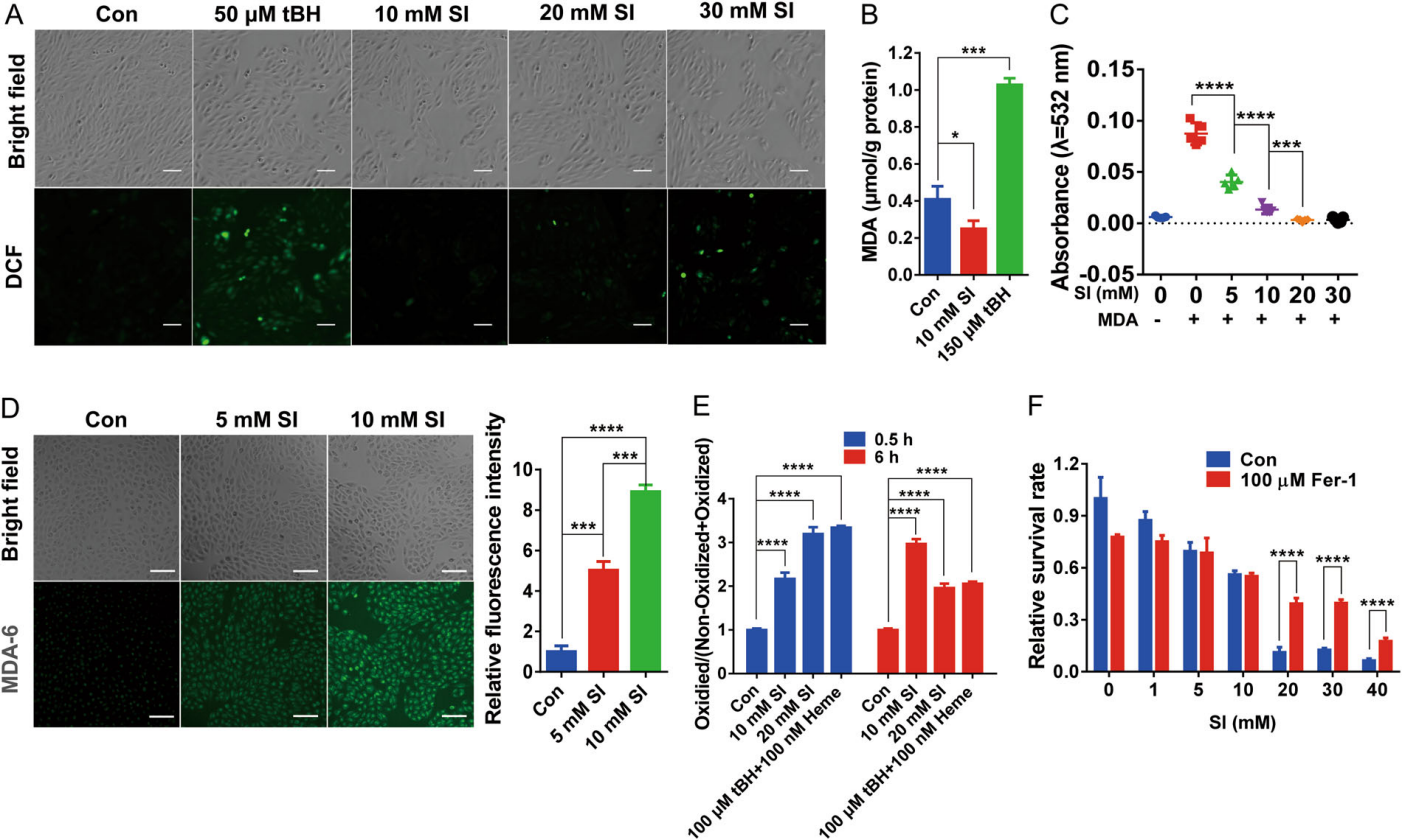
SI promotes ROS generation and oxidative damage of lipids in ARPE-19 cells. **A** Concentration dependent induction of ROS by SI in ARPE-19 cells. Cells were stained with the green fluorescent ROS probe DCFH-DA for 30 min and then exposed to 10, 20, or 30 mM NaIO_3_ or 50 μM tBH as a positive control for 30 min. Scale bar: 100 μm. **B** Detection of MDA content by TBA test in cells treated for 24 h with 10 mM NaIO_3_ or 150 μM tBH. MDA level was expressed in μmol/g protein (*n* = 3). **C** In vitro reaction between SI and MDA. The residual MDA level was detected by the TBA test after 50 μM MDA was incubated with indicated concentrations of NaIO_3_ for 30 min at 37 °C (*n* = 4). **D** Detection of MDA content by MDA-6. ARPE-19 cells seeded in a 96-well plate were treated with or without 5 mM or 10 mM NaIO_3_ for 2 h followed by washing with PBS and staining with 10 μM MDA-6. Left: Fluorescence images of cells stained with MDA-6. Scale bar: 100 μm. Right: Quantification of fluorescence intensity of fluorescence images. **E** Peroxidation of lipids analyzed by BODIPY 581/591 C11. APRE-19 cells were preloaded with the probe BODIPY 581/591 C11 for 30 min followed by treatment with SI or tBH and heme at indicated concentrations for 0.5 or 6 h. Fluorescence of probe in oxidized (488/520 nm) and non-oxidized (575/600 nm) forms were monitored. The ratio of the oxidized BODIPY 581/591 C11 to the total BODIPY 581/591 C11 (oxidized plus non-oxidized) was calculated to represent the extent of lipid peroxidation (*n* = 6). **F** The protective role of Fer-1 for SI-induced cell death. ARPE-19 cells pretreated with or without 100 μM Fer-1 for 24 h were incubated with indicated concentrations of SI for an additional 24 h. The survival rate of cells was measured by the CCK-8 method (*n* = 4).

Besides iron availability, ferroptosis also relies on ROS generation and lipid peroxidation initiated by the Fenton reaction. To monitor ROS generation in ARPE-19 cells under SI treatment, the fluorescent ROS probe DCFH-DA was incubated with cells before SI treatment. Treatment with from 10 to 30 mM SI produced a concentration-dependent increase in ROS by 30 min although the ROS levels were less than the positive control cells treated with 50 μM tBH for 30 min (Fig. 4A). The increased labile iron and ROS suggest that SI might induce lipid peroxidation that is usually accompanied by increasing malondialdehyde (MDA). Surprisingly, the MDA level of ARPE-19 cells did not increase after 24 h of SI treatment although it did increase with tBH treatment (Fig. 4B). Further investigation by incubating MDA with SI indicates that SI eliminates MDA in 30 min, probably by an extended oxidation reaction^38^ (Fig. 4C). Therefore, we used MDA-6, a more sensitive MDA probe that has recently been developed on the basis of photoinduced electron transfer (PET) mechanism^39,40^, to monitor MDA production in living ARPE-19 cells under SI treatments. We found that treatments with 5 mM or 10 mM SI for 2 h caused a significant increase in MDA production (Fig. 4D). In addition, we used redox-sensitive dye BODIPY 581/591 C11 to determine if SI induced lipid peroxidation. Treating BODIPY-loaded cells with SI for 0.5 or 6 h increased lipid peroxidation as measured by an increased rate of BODIPY 581/591 C11 oxidation (Fig. 4E). We also found that SI was potentially destructive to the plasma membrane by oxidizing unsaturated fatty acid directly (Fig. S3). The idea that SI-induced oxidative damage of lipids played crucial roles in cell death was supported by the fact that Fer-1, a lipid antioxidant, and a ferroptosis inhibitor^22^, significantly decreased cell death induced by 24 h of SI treatments (Fig. 4F, Fig. S2). These studies suggest that SI-induced oxidative damage of lipids, directly and indirectly, contributed to cell death.

### SI disrupts redox balance by depleting reduced glutathione and cysteine in ARPE-19 cells

**Fig. 5:**
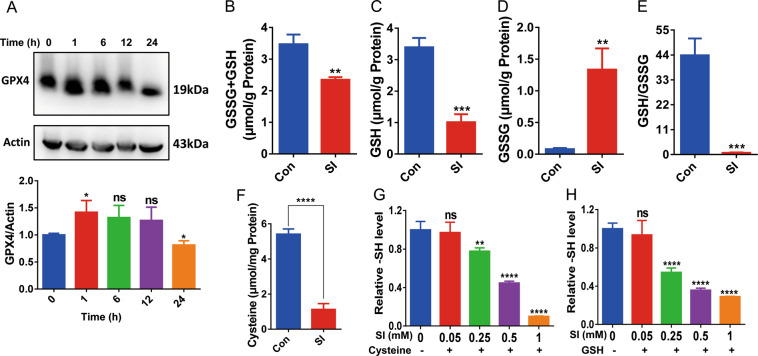
SI reacts directly with cysteine and GSH in ARPE-19 cells. **A** Upper panel: Western blotting detection of GPX4 expression in cells treated with 10 mM NaIO_3_ for the indicated time. Lower panel: the quantifications of three repeats from the upper panel. **B**–**E** The glutathione levels in ARPE-19 cells treated for 24 h with or without 10 mM NaIO_3_. **B** Total glutathione levels; **C** reductive glutathione levels; **D** oxidative glutathione levels; and **E** ratios of reductive to oxidative glutathione levels (*n* = 3). **F** The concentration of cysteine in ARPE-19 cells with or without 10 mM NaIO_3_ treatment for 24 h (*n* = 3). **G**, **H** In vitro reaction between SI and cysteine or GSH. 10 mM cysteine (**G**) or 10 mM GSH (**H**) was incubated with NaIO_3_ at indicated concentrations for 10 min at 37 °C. The residual sulfhydryl was assayed using a cysteine assay kit (*n* = 4).

GSH-dependent reduction of lipid hydroperoxides to lipid alcohols by glutathione peroxidase 4 (GPX4) provides the primary cytoprotection against ferroptosis^20,41^. To test whether SI induces oxidative damage of lipids through downregulating GPX4, GPX4 protein level was monitored by western blotting in SI-treated ARPE-19 cells (Fig. 5A). However, there was only a minor decrease in GPX4 after 24 h of SI treatment. These results suggest that there are more important factors contributing to SI-induced oxidative damage of lipids. Indeed, the total intracellular glutathione level was decreased about one-third by SI treatment (Fig. 5B). More importantly, SI treatment resulted in the decrease of reduced glutathione (GSH) by more than threefold (Fig. 5C) and about a tenfold increase of oxidized glutathione (GSSG) (Fig. 5D). These changes caused a dramatic decrease in the intracellular GSH/GSSG ratio (Fig. 5E). Because cysteine is generally the limiting amino acid for GSH synthesis, the level of intracellular cysteine was also monitored. SI treatment decreased by about fivefold in intracellular cysteine level (Fig. 5F). Indeed, the incubation of SI with cysteine or GSH resulted in the oxidation of a thiol group (Fig. 5G, H). These data suggest that SI treatment on ARPE-19 cells depleted intracellular GSH and cysteine, which induced ferroptosis.

### SI enhances the release of sequestered iron in ARPE-19 cells

**Fig. 6:**
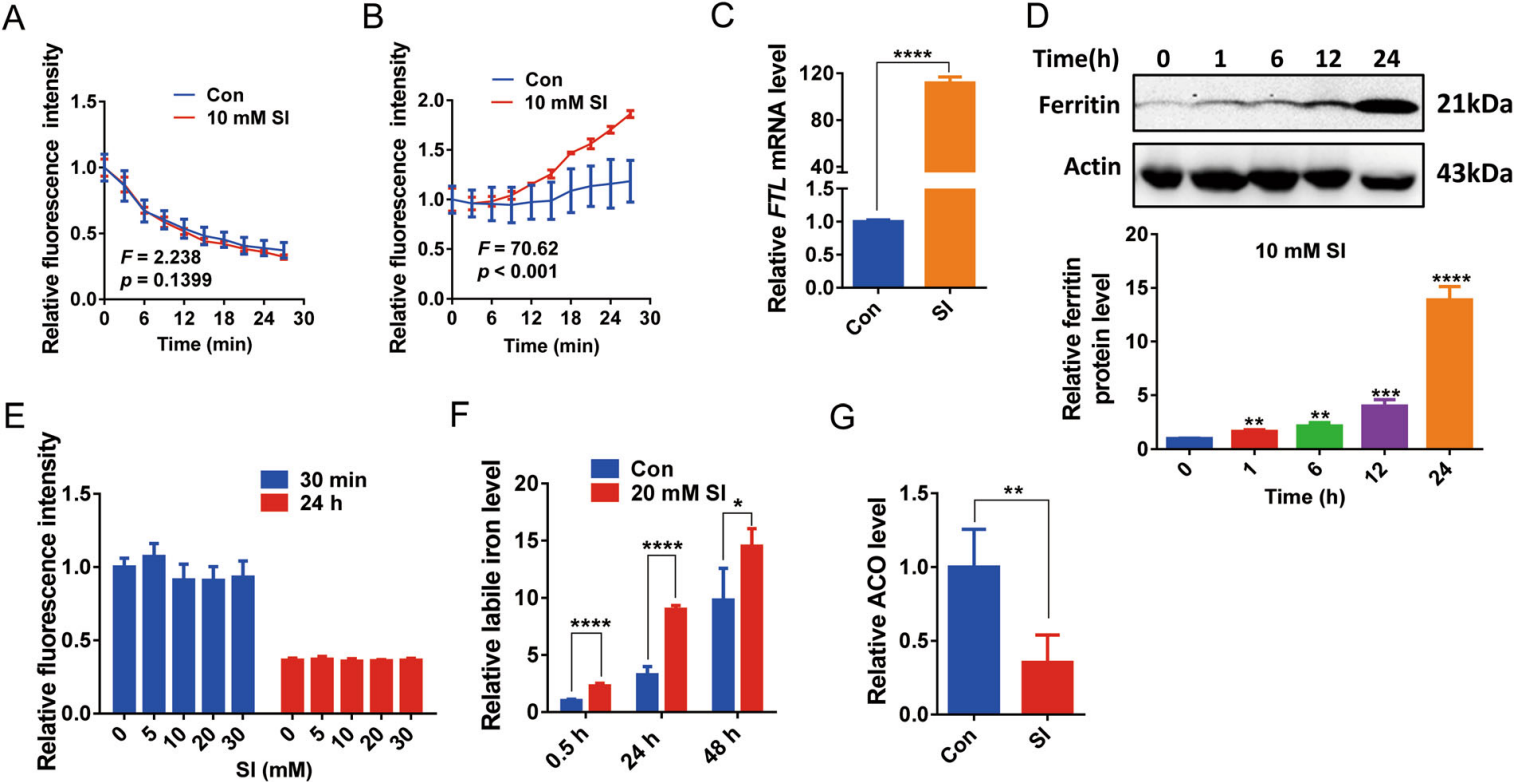
SI induces the increase of labile iron by promoting the release of sequestered iron in ARPE-19 cells. **A**, **B** Ferrous iron influx (**A**) or efflux (**B**) in SI-treated ARPE-19 cells was indicated by the quenching and reversing of calcein fluorescence, a marker of the intracellular labile iron level. The relative fluorescence intensity represented the ratios of the fluorescence intensity of the samples at the indicated time relative to its initial fluorescence intensity. *F* and *P* values were calculated by ordinary two-way ANOVA (*n* = 4). **C** Quantitative real-time PCR analysis of ferritin (ferritin light chain, FTL) mRNA levels in ARPE-19 cells treated with or without 10 mM NaIO_3_ for 24 h (*n* = 3). **D** Upper panel: Western blots of ferritin in ARPE-19 cells incubated with 10 mM NaIO_3_ for the indicated time. Lower panel: the quantifications of three repeats from the upper panel (*n* = 3). **E** SI did not promote the release of free iron in ferritin from the horse spleen. Totally, 0.16 mg/ml ferritin from the horse spleen was treated with NaIO_3_ at the indicated concentrations for 30 min or 24 h and the level of free iron in ferritin solution was determined by calcein fluorescence quenching (*n* = 5). **F** SI promoted the release of free iron in the ARPE-19 cell lysate. ARPE-19 cell lysate was treated with 20 mM NaIO_3_ for 30 min, 24 h, or 48 h and the level of free iron in ferritin solution was determined by calcein fluorescence quenching (*n* = 4). **G** The activity of aconitase (ACO) in the ARPE-19 cell-free lysate treated with or without 10 mM NaIO_3_ for 10 min (*n* = 4).

It is unclear how SI treatment caused the dramatic increase of intracellular labile iron content. To investigate whether SI promotes iron influx or prevents iron efflux in ARPE-19 cells, we examined iron uptake. When cells were incubated in the presence of 1 mM ferrous iron, there was a steady intracellular fluorescence quenching at a similar rate in both control cells and SI-treated cells, suggesting that SI treatment did not promote iron influx (Fig. 6A). After the ferrous iron perfusion, 1 mM DFO was added into medium to chelate extracellular iron and the calcein fluorescence was measured to monitor iron efflux. Surprisingly, SI-treated cells demonstrated a faster increase of calcein fluorescence than control cells, indicating that SI treatment enhanced iron efflux (Fig. 6B). Since iron transport can be enhanced by upregulation of the transferrin receptor^42^, we investigated the level of the transferrin receptor under SI treatment and found it was not altered (Fig. S4). Therefore, the increased intracellular labile iron induced by SI treatment is not likely caused by the alteration of iron uptake. Because ferritin is the major iron storage protein in cells, we analyzed the effects of SI treatment on ferritin expression. SI treatment caused significant increases of both ferritin light chain (FTL) mRNA and protein levels (Fig. 6C, D). These results also indicate that SI did not cause the insufficiency of ferritin for iron storage.

It has been suggested that some reductants cause the release of iron from ferritin^43,44^. To investigate whether SI, as a strong oxidant, causes the release of iron from ferritin as well, we tested if the incubation of SI with purified ferritin (from horse spleen) increased free iron. As expected, SI treatments for 30 min or 24 h did not promote the release of free iron from ferritin (Fig. 6E). Next, we tested if SI causes free iron release from sequestered sources by monitoring the level of free iron in a cell-free ARPE-19 lysate in the absence or presence of SI. In the absence of SI, the level of free iron increased gradually over time (Fig. 6F, blue bars), probably due to the degradation of iron proteins. However, the cell-free lysate incubated with SI had an accelerated increase of free iron, suggesting that SI promoted iron release from sequestered iron sources (Fig. 6F). Furthermore, we monitored the aconitase activity of ARPE-19 cell-free lysate with or without SI because aconitase is an iron–sulfur cluster protein involved in iron homeostasis^45^ and the loss of iron inhibits its activity^46,47^. We found that SI significantly decreased the activity of aconitase in the cell-free lysate (Fig. 6G). These data demonstrate that the increase of labile iron is not caused by iron traffic, but by the release of sequestered irons from iron complexes (see “Discussion”).

## Discussion

Although many RCD pathways have been characterized recently, they were historically classified into three different types, including apoptotic (type I), autophagic (type II), and necrotic (type III) cell death^16^. Necroptosis is a form of type III cell death that crucially requires the sequential activation of RIPK3 and mixed lineage kinase domain-like pseudokinase (MLKL) upon signal initiation by specific death receptors^48,49^. A recent study showed that SI treatment results in RIPK3 accumulation and aggregation in RPE cells in vivo and in vitro^15^. And the inhibition of RIPK1, an activator of RIPK3, by Nec-1 decreased SI-induced RPE cell death^15^. This study suggested that the initial steps of necroptosis were induced by SI. However, it was reported that Nec-1 had off-target effects in ferroptosis indicating that the cytoprotective effects of Nec-1 are not necessarily relied on necroptosis inhibition^19^. In this study, we found that the inhibition of MLKL, whose activation is essential to the execution of necroptosis^49,50^, with NSA did not prevent SI-induced cell death (Fig. 2A). In addition, when ARPE-19 cells were treated with SI, we did not find the enhancement of MLKL phosphorylation, usually observed during necroptosis^48^ (Fig. 2C). Our data raise the question if other necrotic pathways play roles in the SI-induced death of RPE cells.

Ferroptosis has been intensely researched in more than a dozen forms of necrotic cell death, and recognized as a form of RCD with severe lipid peroxidation initiated by ROS and iron^16,51^. Strikingly, we observed more than 40 times of increase in intracellular labile iron level after 24 h treatment of 10 mM NaIO_3_, suggesting ferroptosis contribute to SI-induced cell death (Fig. 3A, B). To understand how labile iron was elevated, we evaluated the effects of SI treatment on the influx and efflux of iron. Our data indicated that SI treatment did not alter the influx of iron while increased the efflux (Fig. 6A, B). Therefore, the iron traffic could not contribute to the increase of labile iron level under SI treatment and it had to be from sequestered iron in the cell. Together with the results showing that SI oxidizes cysteine (Fig. 5F, G) and promotes iron release from cells lysate (Fig. 6F), we propose that SI increases intracellular labile iron level probably by the oxidation of Fe–S proteins in which [Fe–S] clusters are linked to cysteine residues. This hypothesis is supported by the observation that SI significantly decreases the activity of aconitase, which is an iron–sulfur cluster protein complex (Fig. 6G). The process of ferroptosis relies on lipid peroxidation^16,51^. To verify that SI treatment-induced ferroptosis in RPE cells, we tried to evaluate the effects of SI treatment on lipid peroxidation by measuring MDA levels with thiobarbituric acid (TBA). However, we did not observe any increase in MDA-TBA adduct under SI treatment (Fig. 4B). Because aldehydes are usually reactive toward nucleophilic substitution, we suspected that MDA produced could be eliminated by SI. In fact, the extracellular experiment indicated that SI oxidizes MDA directly (Fig. 4C). It suggested that if SI did induce MDA production, a more sensitive method is required for its detection. Indeed, by utilizing a PET mechanism-based fluorescent probe MDA-6, we successfully monitored the change of MDA level in living cells in the presence of SI (Fig. 4D). We were also able to verify the SI-induced lipid peroxidation by using BODIPY 581/591 C11 fluorescent probe (Fig. 4E). In addition, observations of the rupture of the plasma membrane (Fig. 1B, D, E), the increase of labile iron level (Fig. 3A, B) and ROS generation (Fig. 4A), and the protective roles of Fer-1 (Figs. 4F and S2) and DFO (Figs. 3E and S2) indicate that there is ferroptosis induced by SI treatment.

In conclusion, our results indicate that SI induces ferroptosis through the combination of multiple biological and chemical events in ARPE-19 cells. The molecular mechanism of SI-induced ferroptosis relies on the extensive oxidation of thiol groups of cysteine and GSH which in turn promotes the release of labile iron and ROS generation and prevents the scavenging of lipid peroxide by the GSH-dependent enzyme GPX4. Our observations of SI-induced ferroptosis in vitro using ARPE-19 cells and primary mouse RPE cells may not be relevant in vivo. However, we suggest that ferroptosis is an important factor to be evaluated in SI-induced retinal degeneration models. Therefore, key ferroptosis factors including lipid peroxidation, GSH level, ROS generation, and iron availability need to be further addressed in rodent models treated with SI. The protective role of ferroptosis inhibitors in retinal degeneration should also be evaluated in vivo. Additionally, considering the reactivity of SI toward cysteine and GSH, it can be applied as an alternative inducer of ferroptosis in other circumstances.

## Materials and methods

### Reagents and antibodies

The following reagents are obtained commercially: Pre-stained protein marker (10–170 kD) and Tween-20 were from Beyotime Biotechnology (Shanghai, China). Necrostatin-1 (Nec-1), Necrosulfonamide (NSA), Z-VAD-FMK (Z-VAD) and Smac mimetic SM-164, Ferrostatin-1 (Fer-1) were from APExBIO (Houston, USA). GSK'872 was from MedChemExpress (New Jersey, USA). Recombinant Human TNF-α was from Novoprotein (Suzhou, China). Heme was from Alpha (Zhengzhou, China). Tris (trimethylaminomethane), HEPES and dimethyl sulfoxide (DMSO) were from Sigma-Aldrich (Missouri, USA). Ferric ammonium citrate (FAC) was from Yuanye Bio-Technology Co., Ltd. (Shanghai, China). Fluorescent probe MDA-6 was presented by Professor Bo Zhang and Professor Shiguo Sun from Shihezi University, China. Anti-RIPK1 antibody (ab170192), Anti-RIPK3 antibody (ab56164), Anti-MLKL antibody (ab184718), Anti-MLKL (phosphor-S358) antibody (ab187091) and anti-glutathione peroxidase 4 (GPX4) antibody (ab125066) were purchased from Abcam (Cambridge, UK). Phospho-RIPK1 (Ser 166) (D813A) rabbit mAb #44590 was from Cell Signaling Technology (Danvers, MA, USA); peroxidase affinipure (HRP) goat anti-mouse IgG and peroxidase affinipure (HRP) goat anti-rabbit IgG were from ZEN Bioscience (Chengdu, China). Ferritin rabbit monoclonal antibody and β-actin mouse monoclonal antibody were from Beyotime Biotechnology (Shanghai, China). Transferrin Receptor Monoclonal Antibody (Catalog # 13-6800) was from Thermo Fisher Scientific (Waltham, USA).

### Cell lines and culture conditions

Human RPE (ARPE-19) cells were obtained from American Type Culture Collection (ATCC). HT-29 cells were purchased from Gefan Biotechnology Co., Ltd. SH-SY5Y cells were purchased from the Institute of Cells Biology, Shanghai, China. Jurkat cells were bought from Beyotime Biotechnology, Shanghai, China. ARPE 19 cells and SH-SY5Y cells were cultured in DMEM/F-12 (1:1) medium (HyClone, Utah, USA) supplemented with 2.5 mM l-glutamine, 15 mM HEPES buffer, 10% fetal bovine serum (FBS) (Gibco, NY, USA) and 1% (v/v) penicillin (100 U/ml)/streptomycin (100 μg /ml) (Hyclone, Utah, USA) at 37 °C, 5% CO_2_. HT-29 cells and Jurkat cells were cultured in RPMI-1640 medium (Gibco, Beijing, China) supplemented with 10% FBS and 1% (v/v) penicillin (100 U/ml)/streptomycin (100 μg /ml) (P/S). All cell line cells used in this study were the third to seventh passage after resuscitation. Mouse primary RPE cells were isolated from the eyes of 5-day-old C57BL/6 mice and cultured as previously described^52^.

### Assessment of cell morphology by scanning electron microscope (SEM)

ARPE-19 cells were seeded at a density of ~2 × 10^5^ cells/well in a 6-well plate containing cell climbing slides and cultured at 37 °C with 5% CO_2_. When the cell confluency reached about 75%, the medium was replaced by fresh medium with or without NaIO_3_ (Sigma-Aldrich, Missouri, USA). After 24 h of treatment, cells were fixed with 4.0% glutaraldehyde (Chron Chemicals, Chengdu, China) for 30 min and were dehydrated with 30, 50, 70, 80, 90, 95, and 100% ethanol (Chron Chemicals, Chengdu, China) sequentially at room temperature. Dehydrated cell climber slides were coated and analyzed by SEM (JEOL, Tokyo, Japan).

### Cell counting kit-8 (CCK-8) assay

Cell viability was measured by a CCK-8 kit (Zoman Biotechnology, Beijing, China) based on the dehydrogenase activity detection in viable cells. In brief, after 3 times of washing with phosphate-buffered saline (PBS), ARPE-19 cells in a 96-well plate were incubated with 10 μl CCK-8 solution for 2 h. The amount of formazan dye was measured by detecting the absorbance at a wavelength of 450 nm with a microplate reader (Bio-Rad iMark Microplate Reader, USA).

### LDH leakage assay

The toxicity of SI to ARPE-19 cells was assessed by the leakage of intracellular enzyme LDH into the extracellular medium, which is a hallmark of rupture of plasma membrane^53^. The activity of LDH was measured by LDH cytotoxicity assay kit (Beyotime Biotechnology, Shanghai, China) accordingly to the manufacturer’s protocol. The kit is based on the reduction of 2-piodophenyl-3-p-nitrophenyl-5-phenyl tetrazolium chloride (INT) to formazan by diaphorase in the presence of NADH which is converted from lactate and NAD^+^ by LDH. The absorbance of formazan was measured at 490 nm using a microplate reader (Thermo Fisher Scientific, USA) and the absorbance represented the activity of LDH. In brief, 100 μl ARPE-19 cells were seeded in a 96-well plate with a density of ~2 × 10^5^ cells/ml and incubated in a humidified atmosphere containing 5% CO_2_ at 37 °C. After the cell confluency reached about 75%, cells were exposed to indicated concentrations of SI for 24 h. The cytotoxicity of SI was evaluated by the percentage of LDH release, which was calculated by the LDH activity in the extracellular medium divided by the total enzyme activity (extracellular LDH and intracellular LDH).

### Measurement of intracellular ROS

The fluorescent probe 2′,7′-Dichlorodihydrofluorescein diacetate (DCFH-DA) (Sigma-Aldrich, Missouri, USA) was used to measure intracellular ROS level. Briefly, 24 h after seeding (at 70–80% density), ARPE-19 cells were washed with KRPH buffer (Gibco, Gaithersburg, USA), treated with 10 μM DCFH-DA (diluted in KRPH buffer) for 30 min (at 37 °C, 5% CO_2_). Then cells were washed with KRPH buffer again and treated with tBH (Sigma-Aldrich, Missouri, USA) or SI (diluted in KRPH buffer) at 37 °C for 30 min. Fluorescence of DCF resulted from intracellular DCFH-DA hydrolysis was measured using a fluorescence microscope (Leica DMi 8, Leica Microsystems, Germany).

### Hoechst 33342 and PI staining assay

To detect the changes in nuclear morphology (nuclear condensation and fragmentation) of ARPE-19 cells after SI treatment, Hoechst 33342/PI staining was performed. ARPE-19 cells were seeded in a 6-well plate in a volume of 2.0 mL (~2 × 10^5^ cells/well) and incubated in a humidified atmosphere containing 5% CO_2_ at 37 °C for 24 h. Then, cells were treated with 10 mM NaIO_3_ for 24 h followed by washing with KRPH buffer and staining with 36 µg/ml Hoechst 33342 (Solarbio, Beijing, China) and 36 µg/ml PI (Solarbio, Beijing, China) for 20 min at 4 °C. The stained cells were examined by fluorescence microscope (Leica DMi 8, Leica Microsystems, Germany).

### Annexin V-FITC / PI staining

Annexin V-FITC/PI staining of ARPE-19 cells was performed by using a commercially Annexin V-FITC/PI Apoptosis Detection Kit (Beyotime Biotechnology, Shanghai, China) according to the manufacturer’s instructions. Briefly, ARPE-19 cells were seeded in a 6-well plate and cultured at 37 °C with 5% CO_2_ until the cell confluency reached about 75%. Then the cells with or without 10 mM NaIO_3_ treatments for 24 h were harvested by centrifugation and stained with Annexin V-FITC and PI. Next, stained cells were transferred to the flow tubes and analyzed by FACS Caliber flow cytometer (Becton Dickinson, San Jose, CA, USA) and CellQuest analysis software (BD Biosciences).

### TUNEL assay

One Step TUNEL Apoptosis Assay Kit (Beyotime Biotechnology, Shanghai, China) was used for TUNEL staining according to the manufacturer’s instruction. Briefly, ARPE-19 cells were seeded in a 96-well plate at a density of 8000 cells/well. After 24 h of growth, cells were treated with or without 10 mM NaIO_3_ for indicated time. Cells were then washed by PBS and fixed with 4% paraformaldehyde (Biosharp, Hefei, China) for 30 min. After the permeabilization with 0.3% Triton X-100 (Biofroxx, Einhausen, German) for 5 min, cells were incubated with TUNEL reaction mix at 37 °C for 1 h in the dark. The fluorescence was observed with a fluorescence microscope (Leica DMi 8, Leica Microsystems, Germany).

### MMPs assay

The MMP in ARPE-19 cells was measured by JC-1 (5,5′,6,6′-tetrachloro-1,1′,3,3′-tetraethylbenzimidazolcarbocyanine iodide) probe (Beyotime Biotechnology, Shanghai, China) which generates red or green fluorescence based on the status of MMP^54^. In brief, ARPE-19 cells were seeded in a 6-well plate and cultured at 37 °C with 5% CO_2_ until the cell confluency reached about 75%. The cells with or without 10 mM NaIO_3_ treatment for 24 h were washed with PBS and stained with 10 μg/ml of JC-1 dye in DMEM/F12 media at 37 °C in dark for 20 min. The fluorescence images were taken by fluorescence microscope (Leica DMi 8, Leica Microsystems, Germany).

### Measurement of MDA

The MDA level was assayed using two methods. By the one method, a traditional MDA Assay Kit (Solarbio, Beijing, China) based on TBA test was used according to the manufacturer’s instructions. In brief, ARPE-19 cells were seeded in a 10 cm plate and cultured at 37 °C with 5% CO_2_. When the cell confluency reached about 75%, cells were treated with 10 mM NaIO_3_ or 150 μM tBH for 24 h. The cells after the treatment were collected and lysed in PBS by ultrasonication. The resulting cell-free lysate was centrifuged at a rate of 12,000 × *g* at 4 °C for 10 min, and the supernatant was collected. The MDA in the sample reacted with TBA to form an MDA-TBA adduct. The latter was quantified colorimetrically at 532 nm using a microplate reader (Multiskan GO, Thermo Scientific, USA). The levels of MDA were normalized to protein concentrations determined with Coomassie brilliant blue method (Beyotime Biotechnology, Shanghai, China). For the extracellular reaction of MDA with SI, 100 μl of 50 μM MDA (Solarbio, Beijing, China) was incubated with 100 μl of NaIO_3_ in PBS at indicated concentrations for 30 min at 37 °C. The level of residual MDA was determined by using the MDA detection kit as indicated above.

By another method, The MDA level in ARPE-19 cells was detected with fluorescent probe MDA-6 according to the method of Zhang et al.^39^. In brief, ARPE-19 cells were seeded in a 96-well plate at a density of 8000 cells/well and cultured at 37 °C with 5% CO_2_ until the cell confluency reached about 95%. The cells with or without 2 h of SI treatment were washed with PBS and stained with 10 μM of MDA-6 dye in serum-free DMEM/F12 medium at 37 °C in dark for 30 min. The fluorescence images were taken by fluorescence microscope (Leica DMi 8, Leica Microsystems, Germany) and fluorescence intensities were quantified by Image J software.

### Measurement of lipid peroxidation with BODIPY 581/591 C11 assay

The ability of SI to induce lipid peroxidation was investigated using lipid peroxidation sensor BODIPY 581/591 C11 (Thermo Fisher Scientific, Waltham, USA). BODIPY 581/591 C11 is a lipophilic fluorescent dye for indexing lipid peroxidation in cellular membranes^55,56^. In brief, ARPE-19 cells were seeded in a 96-well plate at density of 8000 cells/well. After 24 h, cells were loaded with 2 μM BODIPY 581/591 C11 for 30 min at 37 °C followed by treatment with or without NaIO_3_ for 0.5 h or 6 h. As a positive control, cells were treated with 100 μM tBH and 100 nM Heme for 0.5 h or 6 h. Then the rates of lipid peroxidation were measured using a Varioskan Flash multimode reader (Thermo Fisher Scientific, USA) with excitation/emission of 495/521 nm for the green signal (oxidized) and 575/600 nm for the red signal (non-oxidized). Oxidation of BODIPY 581/591 C11 is presented as a ratio between green fluorescence (oxidized) and total fluorescence (oxidized plus non-oxidized).

### Measurement of cysteine

The Cysteine level in ARPE-19 cells was assayed by cysteine assay kit (Solarbio, Beijing, China), following the manufacturer’s guidelines. In brief, ARPE-19 cells were seeded in a 10 cm plate and cultured at 37 °C with 5% CO_2_. When the cell confluency reached about 75%, cells were treated with or without 10 mM SI for 24 h followed lysed in PBS. The sulfhydryl of Cysteine in the sample interacted with phosphotungstic acid to produce tungsten blue. The light absorption value of the latter was measured at 600 nm with the microplate reader (Multiskan GO, Thermo Fisher Scientific, USA). The concentration of Cysteine was defined by the standard curve of Cysteine. The Cysteine of the sample was normalized to protein concentration determined as above. Cysteine level was expressed in μmol/mg protein.

### Glutathione assay

GSH and GSSG levels in ARPE-19 cells were detected by GSH and GSSG Assay Kit (Beyotime Biotechnology, Shanghai, China), according to the manufacturer’s instructions. The method of sample collection was the same as that for cysteine measurement as mentioned above. To quantify total glutathione (GSH and GSSG), the GSSG in the sample was reduced to GSH by glutathione reductase, followed by oxidizing by the disulfide reagent of 5,5′-dithio-bis (2-nitrobenzoic acid) (DTNB) to form the yellow derivative 5′-thio-2-nitrobenzoic acid (TNB). The latter was determined at 412 nm using a microplate reader (Multiskan GO, Thermo Fisher Scientific, USA). For the determination of GSSG, endogenous GSH in the sample was removed by GSH scavenger before DTNB oxidization. The content of GSH was calculated by deducting the content of GSSG from the total glutathione. The GSH and GSSG levels of the sample was normalized to protein concentration measured as above. The GSH and GSSG level was expressed in μmol/g protein.

### Measurement of the LIP level

The intracellular labile iron was quantified by monitoring the recovering of calcein fluorescence induced by iron chelators after the calcein fluorescence was quenched by intracellular labile iron^57^. Briefly, ARPE-19 cells in a 96-well plate were loaded with 0.25 μM calcein acetoxymethyl ester (CA-AM; calcein-AM) (Sigma-Aldrich, Missouri, USA) for 30 min at 37 °C. After three times washing with KRPH buffer (Hyclone, Utah, USA), 200 μM membrane-permeable iron chelator PIH (APExBIO, Houston, USA) was added. Fluorescence was recorded at an excitation wavelength of 485 nm and an emission wavelength of 530 nm with a Varioskan Flash multimode reader (Thermo Fisher Scientific, USA) before (baseline) and 30 min after PIH addition. Cells were imaged using a fluorescence microscope (Leica DMi 8, Leica Microsystems, Germany) and counted using the Image J Cell Counter plugin. The increase in fluorescence intensity (Δ*F*) was calculated and normalized to the cell number.

### The iron influx and efflux assay

The ferrous iron traffic of ARPE-19 cells was determined by measuring the quenching or reversing of calcein fluorescence as previously described with some modifications^58–60^. In brief, ARPE-19 cells were seeded in a 96-well plate and grown in a serum-free medium with 10 mM NaIO_3_ for 24 h. Then cells were incubated with 0.25 μM calcein-AM in HEPES buffer (10 mM HEPES, 150 mM NaCl, pH 7.4) for 30 min at 37 °C. For the iron influx, ARPE-19 cells were perfused with 1 mM ferrous sulfate in vitamin C solution (1:44 molar ratio, pH 6.0), then calcein fluorescence signal was recorded using a Varioskan Flash multimode reader (Thermo Fisher, Scientific, USA) at 485 nm excitation and 530 nm emission wavelengths. The fluorescence signal was measured every 3 min for 10 times and normalized to the baseline (Time = 0 min). For the iron efflux assay, ARPE-19 cells in the 96-well plate were washed with HEPES buffer for three times after 30 min incubation with 1 mM ferrous sulfate. Then cells were exposed to 1 mM DFO (APExBIO, Houston, USA), a membrane-impermeable iron chelator, for 30 min at 37 °C to drain irons out to the medium, which was indicated by the increase in calcein fluorescence. The fluorescence signal was recorded as above and normalized to the baseline (Time = 0 min).

### Western blot analysis

After treated as indicated, cells were collected and lysed on ice for 10 min with the lysis buffer, which contained 0.2 M Tris–HCl (pH 6.8), 3 mM SDS (Aladdin, Shanghai, China), 2% glycerol (Kelong, Chengdu, China), 0.2% mercaptoethanol (Sigma-Aldrich, Missouri, USA), 60 μM bromophenol blue (Solarbio, Beijing, China), 1 mM PMSF (Phenylmethyl sulfonyl fluoride) (Beyotime Biotechnology, Shanghai, China) and 1% protease inhibitor cocktail (APExBIO, Houston, USA). The proteins were separated on 12% or 15% SDS-PAGE gels and transferred to PVDF (polyvinylidene difluoride) membranes (0.22 μM) (Millipore, Massachusetts, USA). After blocking for 1 h at 4 °C with 8% nonfat milk (Beyotime Biotechnology, Shanghai, China) in TTBS (150 mM NaCl, 20 mM Tris-HCl, 0.1% Tween-20, pH 7.5), the membranes were incubated with the primary antibodies for 6 h at 4 °C and with HRP-conjugated secondary antibody goat anti-rabbit IgG (H&L) or rabbit anti-mouse IgG (H&L) for 1 h at 4 °C. The protein bands were visualized using the ultra-sensitive ECL chemiluminescence kit (Beyotime Biotechnology, Shanghai, China) by UNIVERSAL HOOD II gel imager (Bio-Rad, USA). The images were analyzed by using image lab 6.0 software (BIO-RAD, USA).

### Quantitative real-time PCR

After the treatment, total RNA from ARPE-19 cells was extracted using Beyozol (Beyotime Biotechnology, Shanghai, China) according to the manufacturer’s instructions. cDNA was synthesized from 600 ng total RNA using the BeyoRT^™^ II cDNA synthesis Kit (with gDNA Eraser) (Beyotime Biotechnology, Shanghai, China) according to the manufacturer’s protocol. Quantitative real-time PCR was performed using BeyoFast^™^ SYBR Green qPCR Mix (2×, Low ROX) (Beyotime Biotechnology, Shanghai, China) in a CFX96 Touch real-time quantitative PCR instrument (Bio-Rad, USA) and CFX96 real-time system (Bio-Rad Laboratories, USA). Primers used for quantitative real-time PCR were *FTL*: forward 5′-AAA GCT GAA CCA GGC CCT TT-3′, reverse 5′-GAA GAG ATA CTC GCC CAG CC-3′; *ACTB* (actin beta): forward 5′-CCT GGG CAT GGA GTC CTG TG-3′, reverse 5′-AGG GGC CGG ACT CGT CAT AC. The reaction program was as follows: 42 °C preheating for 5 min, 95 °C pre denaturing for 10 s, 94 °C denaturing for 5S, 58 °C annealings for 30 s, 72 °C for 15 s, 40 cycles. The relative mRNA expression level was calculated by the 2^−ΔΔCt^ method and ACTB was used as the housekeeping gene.

### The extracellular reaction of ferritin with SI

The direct effect of SI on the releasing of free iron from ferritin was determined by calcein fluorescence quenching assay. Briefly, 0.16 mg/ml ferritin from horse spleen (Sigma-Aldrich, Missouri, USA) was incubated with 0, 5, 10, 20, and 30 mM NaIO_3_ for 30 min or 24 h at 37 °C. Then each sample was incubated with 6 μl of 0.2 mM calcein (APExBIO, Houston, USA) solution at 37 °C for 30 min. The calcein fluorescence signal was recorded as above.

### Monitoring free iron in a cell-free ARPE-19 lysate

About 5 × 10^7^ ARPE-19 cells from five 10 cm dishes were collected by centrifugation for 3 min at 1500 rpm. Cells were lysed in 1250 μl PBS by ultrasonication and centrifuged for 10 min at 4 °C and 12,000 × *g*. The supernatant was collected as the cell-free lysate. To determine whether SI causes the release of free iron from the cell-free lysates, 100 μl lysate and 6 μl of 0.2 mM calcein, with or without 30 μl 100 mM NaIO_3_ and 20 μl PBS, were mixed in the wells of a 96-well plate and incubated at 37 °C for the indicated time. The releasing of free irons in the cell-free lysate was monitored by calcein fluorescence quenching assay as indicated above.

### Aconitase activity assay

ARPE-19 cells were lysed in PBS and 400 μl of the cell-free lysate (0.53 mg/ml) was mixed with 100 μl of 100 mM NaIO_3_ or 100 μl of solvent alone (PBS). After incubation at 37 °C for 10 min, NaIO_3_ was removed by ultrafiltration using ultrafiltration filters with a 50 kDa molecular weight cutoff (Millipore, Massachusetts, USA). Aconitase (ACO) activity assay kit (Solarbio, Beijing, China) was used to detect the ACO activity of ARPE-19 cell-free lysate by following the manufacturer’s instructions.

### The extracellular reaction of Cysteine and GSH with SI

Totally, 20 μl 10 mM Cysteine (Solarbio, Beijing, China) in PBS or 20 μl 10 mM GSH (Beyotime Biotechnology, Shanghai, China) in PBS was incubated with 60 μl of 0, 0.05, 0.25, 0.5, or 1 mM NaIO_3_ in PBS for 10 min at 37 °C. The oxidation of the thiol group in Cysteine or GSH was monitored with a Cysteine assay kit as indicated above.

### The extracellular reaction of linoleic acid with SI

The oxidation of linoleic acid by SI was assayed by ultraviolet absorption spectroscopy^61,62^. Briefly, 10 μl of linoleic acid at ≥99% purity (Sigma-Aldrich, Missouri, USA) was mixed with 490 μl of NaIO_3_ in PBS at indicated concentrations followed by 30 min of incubation at 37 °C. All tubes were filled with nitrogen to prevent oxygen in the air from oxidizing linoleic acid. After the reaction, 900 μl of purified chloroform (Knowles, Chengdu, China) was added to extract the linoleic acid. The absorbance of linoleic acid was recorded with UV–visible spectrophotometer (UV2550, Shimazu, Japan) in the wavelength range of 260 nm-330 nm.

### Statistical analysis

All experiments were repeated at least three times. Statistical analyses were performed using GraphPad Prism software (Version 6.0, USA). The data were expressed as mean ± SD and analyzed using unpaired *t* test or ordinary two-way ANOVA. Statistical significance was defined as a *p* value of >0.05, <0.05, <0.01, <0.001, and <0.0001 was marked as ns, *, **, ***, and ****, respectively.

## Supplementary information

Supplementary figure captions

Figure S1 Optical microscopy of ARPE-19 cells under SI treatments.

Figure S2 The protective role of Fer-1, PIH and DFO for SI induced death of mouse primary RPE cells.

Figure S3 In vitro reaction between SI and linoleic acid (La)

Figure S4 Transferrin receptor levels in ARPE-19 cells under SI treatments
