# Supplementary material for: Sodium iodate induces ferroptosis in human retinal pigment epithelium ARPE-19 cells
Source: Cell Death Dis. 2021 Mar 3;12(3):230. doi: 10.1038/s41419-021-03520-2 (PMC7930128; doi:10.1038/s41419-021-03520-2)
Supplement: Supplementary file 1 — Supplementary figure captions [file 41419_2021_3520_MOESM1_ESM.docx]

**Supplementary figure captions**

**Figure S1 Optical microscopy of ARPE-19 cells under SI treatments.**

ARPE-19 cells were cultured with or without NaIO_3_ at indicated concentrations for 24 h. After the treatments, the bright field microscopy images were recorded. Scale bar: 100 μm.

**Figure S2 The protective role of Fer-1, PIH and DFO for SI induced death of mouse primary RPE cells.**

The mouse primary RPE cells were pretreated with 50 μM Fer-1, 25 μM PIH or 100 μM DFO or solvent alone (DMSO) for 24 h prior to co-treatment with 20 mM NaIO_3_ for 24 h. A. The bright field microscopy images were recorded after respective treatments. Note that black dots in the field are pigment typically produced by primary RPE cells of C57BL/6 mouse. Scale bar: 50 μm. B. Cell viabilities after respective treatments were determined by CCK assays (n=4).

**Figure S3 *In vitro* reaction between SI and linoleic acid (La).**

2% v/v La was reacted with indicated concentrations of NaIO_3_ for 30 min at 37 °C and the absorbance was measured between 260 nm~330 nm. (n=4).

**Figure S4 Transferrin receptor levels in ARPE-19 cells under SI treatments.**

Upper panel: Western blots of transferrin receptor in ARPE-19 cells incubated with 10 mM NaIO_3_ for indicated time. Lower panel: The quantifications of four repeats of western blots.
