# Supplementary figures and images for: Sodium iodate induces ferroptosis in human retinal pigment epithelium ARPE-19 cells
Source: Cell Death Dis. 2021 Mar 3;12(3):230. doi: 10.1038/s41419-021-03520-2 (PMC7930128; doi:10.1038/s41419-021-03520-2)

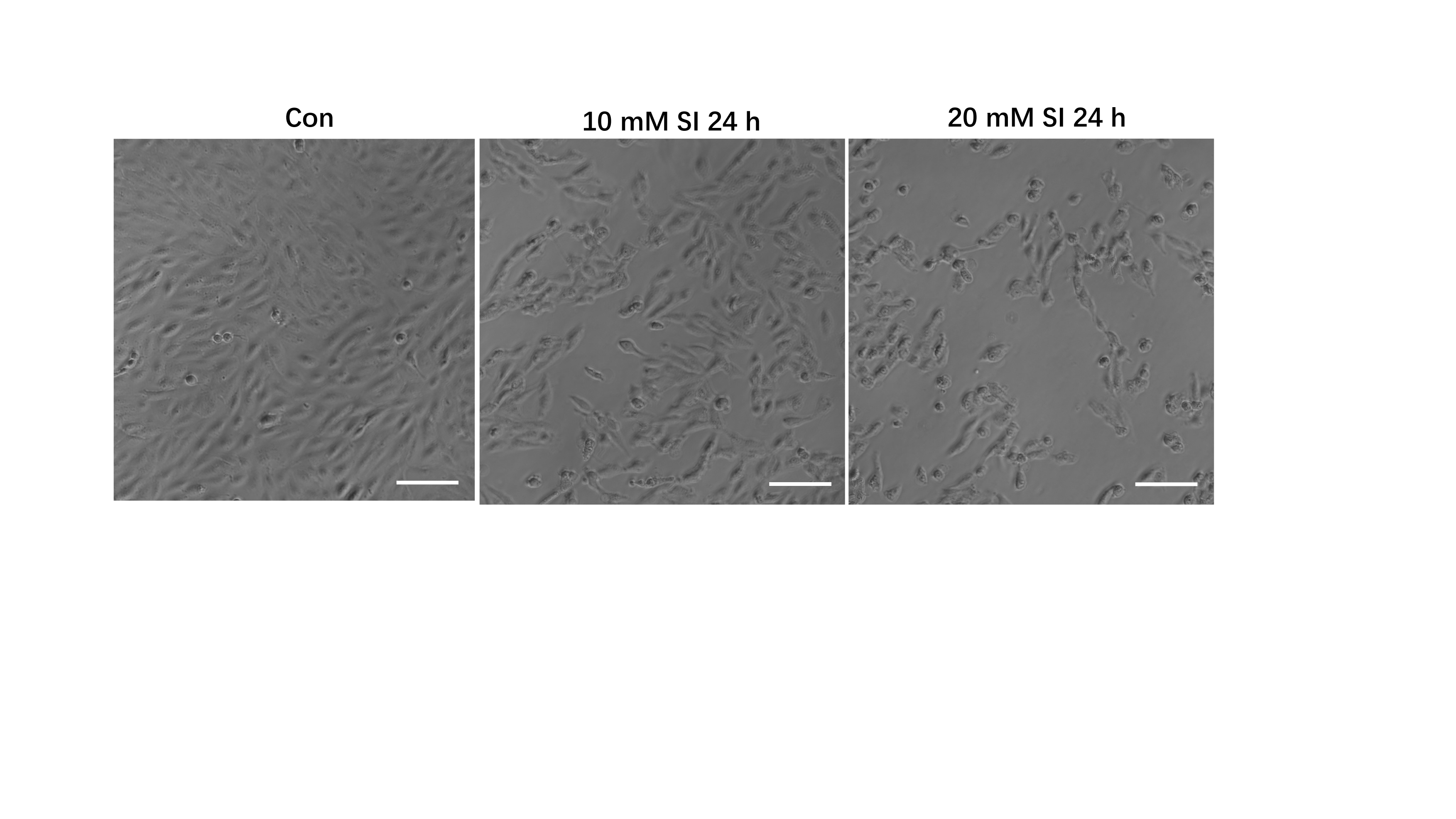

Supplement: Supplementary file 2 — Figure S1 Optical microscopy of ARPE-19 cells under SI treatments. [file 41419_2021_3520_MOESM2_ESM.tif]

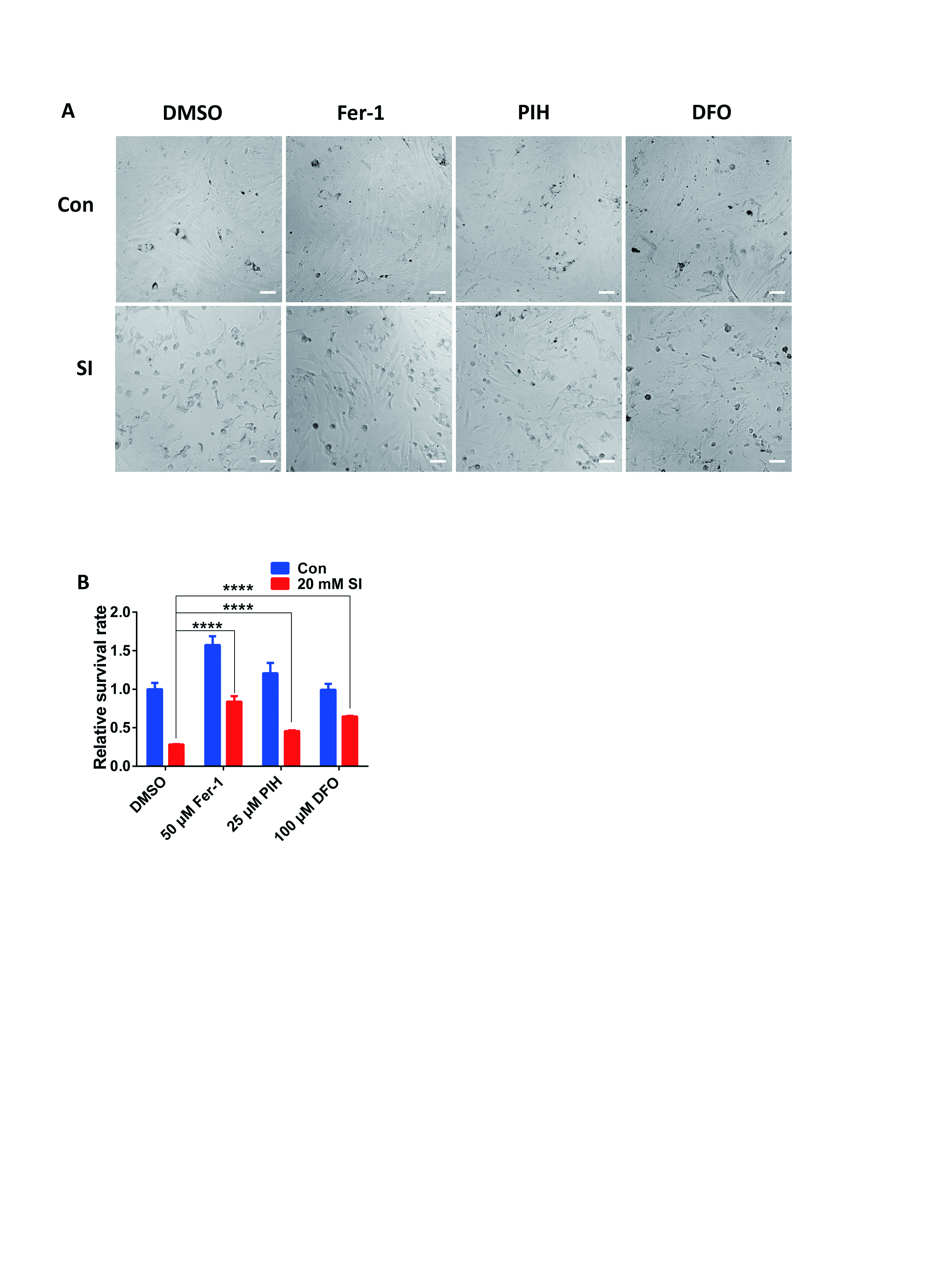

Supplement: Supplementary file 3 — Figure S2 The protective role of Fer-1, PIH and DFO for SI induced death of mouse primary RPE cells. [file 41419_2021_3520_MOESM3_ESM.tif]
